# Metabolism-Driven High-Throughput Cancer Identification with GLUT5-Specific Molecular Probes

**DOI:** 10.3390/bios8020039

**Published:** 2018-04-10

**Authors:** Srinivas Kannan, Vagarshak V. Begoyan, Joseph R. Fedie, Shuai Xia, Łukasz J. Weseliński, Marina Tanasova, Smitha Rao

**Affiliations:** 1 Department of Biomedical Engineering, Michigan Technological University, Houghton, MI 49931, USA; skannan1@mtu.edu; 2 Department of Chemistry, Michigan Technological University, Houghton, MI 49931, USA; vbegoyan@mtu.edu (V.V.B.); jrfedie@mtu.edu (J.R.F.); shuaix@mtu.edu (S.X.); lweselin@mtu.edu (Ł.J.W.)

**Keywords:** breast cancer, fructose mimics, glucose transporters, GLUT5

## Abstract

Point-of-care applications rely on biomedical sensors to enable rapid detection with high sensitivity and selectivity. Despite advances in sensor development, there are challenges in cancer diagnostics. Detection of biomarkers, cell receptors, circulating tumor cells, gene identification, and fluorescent tagging are time-consuming due to the sample preparation and response time involved. Here, we present a novel approach to target the enhanced metabolism in breast cancers for rapid detection using fluorescent imaging. Fluorescent analogs of fructose target the fructose-specific transporter GLUT5 in breast cancers and have limited to no response from normal cells. These analogs demonstrate a marked difference in adenocarcinoma and premalignant cells leading to a novel detection approach. The vastly different uptake kinetics of the analogs yields two unique signatures for each cell type. We used normal breast cells MCF10A, adenocarcinoma cells MCF7, and premalignant cells MCF10AneoT, with hepatocellular carcinoma cells HepG2 as the negative control. Our data indicated that MCF10AneoT and MCF7 cells had an observable difference in response to only one of the analogs. The response, observed as fluorescence intensity, leads to a two-point assessment of the cells in any sample. Since the treatment time is 10 min, there is potential for use in rapid on-site high-throughput diagnostics.

## 1. Introduction

Point-of-care (POC) and POC-healthcare technologies (POCHT) that monitor changes in the intracellular mechanisms to report disease development have gained prominence in clinical and consumer implementations owing to the ease of access to information, low cost, and self-management of health and wellbeing [1,2,3,4,5]. The marked improvement in microfluidics, molecular diagnostics, and nucleic acid chemistries [6,7,8] to identify cancer-relevant biomarkers [9] has led to an increased interest in POC/POCHT for cancer. However, the relatively low concentration of the biomarkers poses constraints on sensing, limiting the identification of the metastatic capability of the tumor, when present. The diagnosis currently relies heavily on various radiological techniques. Despite the advances to improve the resolution of radiological approaches, pathology reports of biopsy samples remain the sole means to identify cancer type and stage [10]. There exist discrepancies in diagnosis for the same sample owing to the highly heterogeneous nature of the tissue, subtle morphological changes, and the interpretation by the observer, particularly when the sample deviates from key criteria used to classify breast tumors [11]. Any approach capable of the identification of cancer cell populations in the heterogeneous environment of the tumor is expected to be based on error-free cancer detection and diagnosis for development of cancer-relevant POC technologies. Within this communication, a metabolism-driven approach for the detection and identification of breast cancers is described. The difference in carbohydrate demands among different cells and cancer subpopulations forms the basis for the approach presented here. 

The long-recognized higher energy demands in cancer cells [12,13] have led to the development of metabolism-based approaches to detect cancer, with ^18^F-2-deoxy-glucose being widely used as a cancer-imaging agent in positron emission tomography (PET) [14]. Until recently, the major efforts in identifying cancer through metabolism changes have focused on targeting glucose uptake and the facilitative glucose transporter GLUT1 [15,16]. However, the global physiological need for glucose and the ubiquitous presence of GLUT1 limits this strategy in sensitivity, selectivity, and specificity [17]. In particular, targeting glucose transport is limited in breast cancers, which are known to exhibit insignificant changes in glucose uptake with respect to their normal counterparts [18,19,20]. Recently, strong links between cancer and enhanced fructose uptake have been established, bringing forth fructose transport as a promising target to identify cancer on the basis of fructose uptake and metabolism [21]. For example, triple-negative breast cancer phenotypes have been reported to exhibit 8–10-fold higher fructose uptake than other phenotypes, with minimal fructose uptake measured for normal breast cells [22,23,24]. Dependence on fructose for growth and progression identified for breast cancers [25,26,27] has justified targeting fructose uptake as means to detect breast cancer. Moreover, it is possible to achieve a high level of specificity by detecting the expression of the fructose-specific transporter GLUT5 present in breast cancer cells, but not normal breast cells [28] using appropriate detection tools.

**Figure 1:**
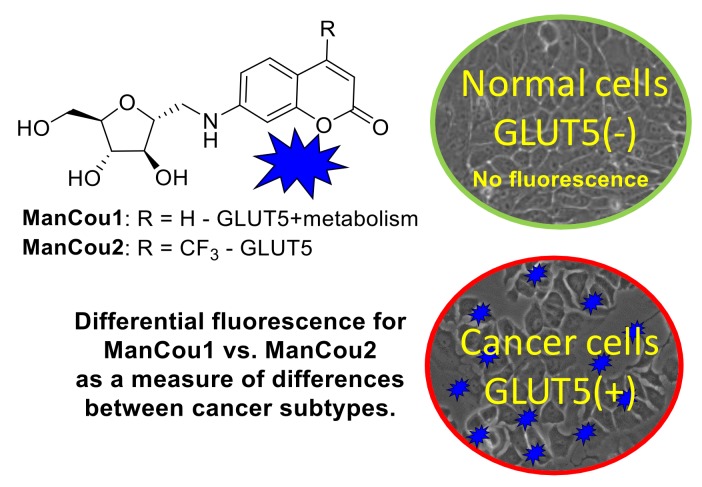
Fluorescence-based identification of breast cancer cells and discrimination of cancer phenotypes through GLUT5 with fluorescent 1-AM-coumarin conjugates ManCou1 and ManCou2.

Targeting GLUTs specifically has led to only a few molecular probes with transporter specificity [16,29]. Fructose transporters have been specifically targeted with fluorescent 7-nitrobenzofurazan (NBD) conjugates of fructose (NBDF) [30]. Aryl conjugates of 1-amino-2,5-deoxy-d-mannitol (1-AM) show high affinity and specificity towards GLUT5 with NBD conjugate of 1-AM working as fluorescent GLUT5 reporter [31,32]. Interestingly, epimers or regio-isomers of NBDM were shown to gain uptake through glucose GLUTs with loss of uptake through the fructose GLUTs [33,34]. While NBDM provided feasibility for discriminating between normal and cancer cells on the basis of the uptake through GLUT5 [32], the probe had limited accumulation in cells (uptake saturation was measured at 50 µM concentration), resulting in the unfavorable background fluorescence. Recently, the family of GLUT5 reporters was extended with coumarin conjugates of 1-AM, providing access to fluorescent probes of different colors [35]. Here we report GLUT5-targeting probes with improved cancer detection based on metabolic profiles. The two probes described differ in their uptake profile and reflect the metabolic capacity of the cell and GLUT5 activity. Considering the recognized differences between cells in their metabolic efficiency as well as GLUT5 expression, the probes enable two-point characterization of cells and allow for discrimination of premalignant cells from differentiated epithelial cells and normal cells under an in vitro setting (Figure 1).

## 2. Materials and Methods

### 2.1. Reagents and Techniques

All reagents were used as received, unless otherwise stated. The 7-aminocoumarin was synthesized according to the reported literature [36] and 7-amino-4-(trifluoromethyl)coumarin was procured from Alfa Aesar. Analytical thin layer chromatography (TLC) was carried out on commercial SiliCycle SiliaPlate^®^ (Ville de Québec, QC, Canada) 0.2 mm F254 plates. Preparative silica chromatography was performed using SiliCycle SiliaFlash^®^ (Ville de Québec, QC, Canada) F60 40–63 μm, 230–400 mesh. Final purification of compounds was achieved with Agilent-1200 HPLC (high-pressure liquid chromatography) using reversed phase semipreparative column (Phenomenex^®^ (Torrance, CA, USA, Luna^®^ 10 µm C18(2) 100 Å, LC Column 100 × 10 mm, Ea)). ^1^H and ^13^C-NMR spectra were recorded at room temperature with a Varian Unity Inova 400 MHz spectrometer. Deuterated methanol (CD_3_OD) was used as a solvent and referenced to the corresponding residual solvent peaks (3.31 and 49.0 ppm, respectively). The following abbreviations are used to indicate the multiplicity: s—singlet; d—doublet; t—triplet; q—quartet; m—multiplet; b—broad signal; app—approximate. The coupling constants are expressed in hertz (Hz). The high-resolution mass spectrometry analysis (HRMS) was carried out with a Thermo Fisher Orbitrap Elite™ Hybrid Ion Trap-Orbitrap Mass Spectrometer at the Chemical Advanced Resolution Methods (ChARM) Laboratory at Michigan Technological University. Analysis of cell fluorescence was carried out with Victor3 fluorescence plate reader (excitation at 385 nm) in a 96-well plate format. Confocal images were taken with Olympus FluoViewTM FV1000 using the FluoView software. Fluorescence imaging was done with EVOS FL Auto inverted microscope.

### 2.2. Synthesis of ManCou Conjugates

General procedure: (2*S*,3*S*,4*S*,5*R*)-3,4-dihydroxy-5-(hydroxymethyl)tetrahydrofuran-2-carbaldehyde [32] (up to 1 mmol) and the corresponding coumarin (0.8 equiv.) were dissolved in methanol (10 mL). The pH of the solutions was adjusted to <6 by acetic acid (1 mL), and NaBH_3_CN was added portionwise to the reaction mixture (3 × 0.8 equiv., every 20–30 min). The reaction solutions were stirred at room temperature for up to 24 h. The mixtures were then concentrated to dryness under reduced pressure and purified by column chromatography on silica gel using methanol in dichloromethane (0–10%) mixtures. The final purification was achieved by semipreparative HPLC using a water–acetonitrile (2–20%) gradient, with 30–40% average yield after final purification. Structures of ManCous were verified through spectroscopic analysis.

*7-((((2R,3S,4S,5R)-3,4-Dihydroxy-5-(hydroxymethyl)tetrahydrofuran-2-yl)methyl)amino)-2H-chromen-2-one (ManCou1)*: ^1^H-NMR (400 MHz, CD_3_OD): *δ*, 7.76–7.36 (d, *J* = 9.2, 1H), 7.32–7.30 (d, *J* = 8.4, 1H), 6.68–6.65 (dd, *J*_1_ = 2.4, *J*_2_ = 8.4, 1H), 6.53 (d, *J* = 2.4, 1H), 6.01–5.99 (d, *J* = 9.2, 1H), 4.02–3.98 (m, 2H), 3.95–3.92 (m, 1H), 3.88–3.85 (m, 1H), 3.73–3.69 (app dd, *J*_1_ = 3.2, *J*_2_ = 12.0, 1H), 3.66–3.61 (app dd, *J*_1_ = 5.6, *J*_2_ = 12.0, 1H), 3.48–3.44 (app dd, *J*_1_ = 3.6, *J*_2_ = 13.6, 1H), 3.38–3.32 (app dd, *J*_1_ = 6.8, *J*_2_ = 13.6, 1H) ppm. ^13^C-NMR (100 MHz, CD_3_OD): *δ*, 164.7, 158.1, 154.5, 146.5, 130.2, 112.3, 110.6, 109.1, 98.0, 85.3, 83.2, 80.3, 78.9, 63.3, 46.2 ppm. HRMS (ESI): *m*/*z* [M + Na]^+^ calc’d for C_15_H_17_NNaO_6_: 330.09539; found 330.09434.

*7-((((2R,3S,4S,5R)-3,4-Dihydroxy-5-(hydroxymethyl)tetrahydrofuran-2-yl)methyl)amino)-4-(trifluoromethyl)-2H-chromen-2-one (ManCou2)*: ^1^H-NMR (400 MHz, CD_3_OD): *δ*, 7.44–7.41 (dd, *J*_1_ = 2.0, *J*_2_ = 9.2, 1H), 6.74–6.71 (dt, *J*_1_ = 2.4, *J*_2_ = 9.2, 1H), 6.60–6.59 (d, *J* = 2.4, 1H), 6.37 (s, 1H), 4.02–3.98 (m, 2H), 3.95–3.92 (m, 1H), 3.89–3.85 (m, 1H), 3.73–3.69 (app dd, *J*_1_ = 3.6, *J*_2_ = 11.6, 1H), 3.66–3.61 (app dd, *J*_1_ = 5.6, *J*_2_ = 12.0, 1H), 3.50–3.46 (app dd, *J*_1_ = 3.6, *J*_2_ = 14.0, 1H), 3.40–3.35 (app dd, *J*_1_ = 6.4, *J*_2_ = 13.6, 1H) ppm. ^13^C-NMR (100 MHz, CD_3_OD): *δ*, 162.2, 158.6, 155.0, 143.3, 143.0, 124.8, 122.1, 112.8, 108.3, 104.1, 98.5, 85.4, 83.2, 80.2, 78.8, 63.3, 46.1 ppm. HRMS (ESI): *m*/*z* [M + H]^+^ calc’d for C_16_H_17_F_3_NO_6_: 376.10082; found 376.09955. 

### 2.3. Tissue Culture

Normal breast cells (MCF 10A/ATCC^®^ CRL-10317™), adenocarcinoma (MCF-7/ATCC^®^ HTB-22™) cells, and hepatocellular carcinoma (HepG2/ATCC^®^ CRL-10317™) cells were procured from American Type Cell Culture. The premalignant breast cancer cells MCF10AneoT was purchased from the Animal Model and Therapeutics Evaluation Core (AMTEC) Karmanos Cancer Institute, Wayne State University. All cells were maintained at 37 °C, at 65% relative humidity, and under 5% CO_2_ in their respective culture mediums (see Appendix A). All cultures were supplemented with 10,000 I.U./mL penicillin and 10,000 μg/mL streptomycin to lower chances of bacterial contamination.

### 2.4. Microplate Uptake and Inhibition Assays

For microplate assays, cells at ~80% confluence were collected and plated in 96-well flat-bottom plates (20,000 cells/well) and allowed to grow for 24 h. Cells were then washed with warm (37 °C) PBS solution, treated with ManCou probes (concentration varies) in PBS and incubated at 37 °C and 5% CO_2_ for 10 min. After incubation, cells were carefully washed with warm PBS (3 × 100 µL). Fluorescent data was immediately collected using the Victor3 plate reader and using WallacTM umbelliferone (excitation 355 nm, emission 460 nm, 1.0 s) protocol. All trials were done in triplicates. The corresponding errors were derived as standard deviation.

Uptake inhibition studies were carried using 96-well plates. Fluorescence of ManCou probes in cells was measured in the presence of varying concentrations of fructose and glucose. For this part, PBS solution containing 20 µM ManCou and the specific concentration of a sugar was prepared and introduced with the cells. In parallel, complete culture media was used to establish the impact of nutrients on ManCou uptake. Cell incubation and data collection were conducted as stated above.

### 2.5. Immunostaining

The GLUT5 (Slc2a5) primary antibody (sc-271055) was obtained from Santa Cruz Biotechnology, Inc. (Dallas, TX, USA) and the secondary antibody (ab6787) was obtained from Abcam (Cambridge, MA, USA). The primary antibody was used with incubation buffer at a 1:200 dilution, while the secondary antibody was used at a dilution of 1:1000, as recommended. All the cells were fixed in 4% paraformaldehyde and blocked for 1 h at room temperature. Incubation for primary and secondary antibodies was 2 h and 1 h, respectively, at room temperature. A PBS rinse was carried out between each step. Imaging was carried out immediately.

### 2.6. Imaging

The individual cell lines in culture were incubated with ManCou1 and 2 for 10 min at a concentration of 20 µM across all tests. The test concentration was established after evaluation of the imaging efficiency within a range of concentrations (5–100 µM). 20-µM concentration was selected as the lowest at which steady fluorescence readout was achieved using a confocal microscope (Olympus FluoViewTM FV1000). Imaging was carried out using EVOS FL autoimaging with the EVOS software. All images were taken at a constant gain setting. From the grayscale images, the corrected total cell fluorescence (CTCF) was calculated as described in our previous work [37]. Briefly, using ImageJ, comparing the average fluorescence with the background of the same image, the fluorescence signal only in the region of interest was obtained as described previously [38]. The process was repeated five times for each image captured to obtain an average fluorescence. The average CTCF obtained was then normalized to the average CTCF obtained for the normal control sample. The normalized average CTCF (with respect to MCF10A) was then plotted in Microsoft Excel. The advantage of using CTCF for images obtained along a z-plane with best focus, as presented by McCloy et al. [38], demonstrates that the information thus obtained eliminates errors due to localized higher levels. 

## 3. Results and Discussion

### 3.1. Design and Evaluation of Fluorescent Fructose Mimics as GLUT5-Specific Probes

**Figure 2:**
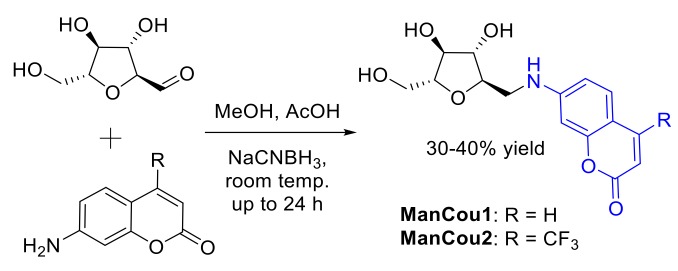
Synthesis of ManCou analogs.

**Figure 3:**
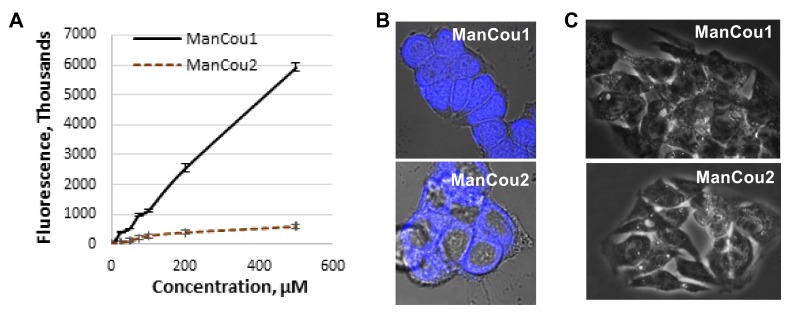
ManCou uptake analysis. (**A**) Concentration-dependent uptake in MCF7 cells in 96-well plate; (**B**) brightfield and fluorescence (DAPI) overlay of confocal Z-stack images of MCF7 with ManCou1 and ManCou2; (**C**) brightfield and fluorescence (DAPI) overlay of confocal Z-stack images of HepG2 cells with ManCou1 and ManCou2. Images taken with 20 µM ManCous at 405 nm excitation and 461 nm emission (60× for MCF7 cells and 40× for HepG2 cells).

Two blue-fluorescent GLUT5 probes were designed and synthesized (Figure 2) on the basis of the established GLUT5 preference for 1-amino-2,5-anhydro-d-mannitol (Man) [31,32] and the capacity of GLUTs to pass coumarin (Cou) fluorophores [39], to enable significant accumulation in the cell through GLUT5. The resulting sugar–coumarin conjugates—“ManCous”—bearing H or CF_3_ at the C4 position of the coumarin ring (ManCou1 and ManCou2, respectively; Figure 3A) were evaluated for the uptake and GLUT5 specificity in cell culture. For this part, MCF7 cells, previously studied for GLUT5-mediated uptake [22,24,30], and HepG2 cells, which lack GLUT5 [40], were used. 

For the initial uptake analysis, MCF7 cells were treated in a 96-well plate with ManCou probes at different concentrations and the cell fluorescence was measured with a fluorescence plate reader. At concentrations exceeding 100 µM, the fluorescence readout for ManCou2 showed signs of saturations and leveled off at 200 µM concentration (Figure 3A). The fluorescence signal from ManCou1 gradually increased and showed no saturation, even at 500 µM concentration. The Z-stack analysis of ManCou-treated cells showed a prominent accumulation of probes inside the cell. While both probes are internalized by the cell, they show a marked difference in cellular distribution. ManCou1 is evenly distributed throughout the cell, including the nucleus, while ManCou2 is localized in the cytosol (Figure 3B). The differences in the cell distribution parallel those in the ManCou uptake profile. The linearity in the uptake of ManCou1 is suggestive of phosphorylation of the probe, leading to the removal of the probe gradient within the cell. On the other hand, saturable uptake for ManCou2 implies lack of phosphorylation and gradient buildup, precluding continuous uptake of the probe. It is then plausible that only phosphorylated species are further taken up by the cell nucleus. While mannitol phosphorylation within the cell is established [41,42,43,44], further analyses are needed to link nuclear accumulation with ManCou phosphorylation. 

**Figure 4:**
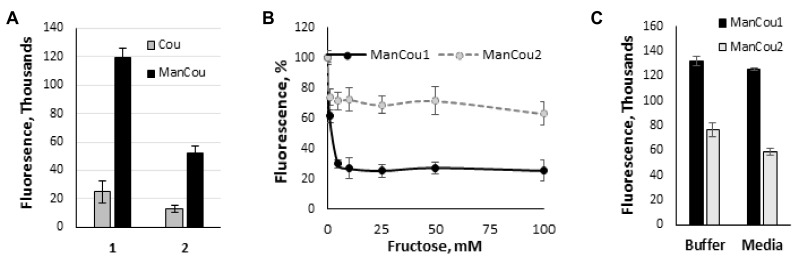
Analysis of sugar impact on ManCou uptake. (**A**) Comparative uptake of ManCous (black bars) vs nonconjugated (grey bars) coumarins; (**B**) fructose-induced inhibition of ManCou uptake; (**C**) ManCou uptake is independent of glucose concentration and is equally effective in the buffer and complete culture medium. All data obtained in 96-well plate format. Each data point constitutes an average of two independent experiments in triplicate. Error bars represent standard deviation.

To further delineate the contribution of the sugar in the uptake of ManCous, a direct comparison of ManCou uptake with that of the nonconjugated coumarin was carried out. At the same concentrations, we have observed six-fold and five-fold lower uptake respectively, for unconjugated coumarins (Cou1 is coumarin of ManCou1, and Cou2 is coumarin of ManCou2; Figure 4A), implying the sugar-driven uptake of ManCous. It also appears that the uptake of ManCou1 is nearly twice as much as ManCou2. The uptake of both ManCous was inhibited by fructose (Figure 4B), although to a greater extent in the case of ManCou1. Glucose, however, had no effect on the uptake, as evident from the equally efficient internalization of the probes in buffer and complete high-glucose culture medium (Figure 4C). Finally, we have tested ManCou uptake in GLUT5-deficient HepG2 cells to delineate any contribution from nonspecific binding to the observed gained fluorescence. While some basal fluorescence has been observed for whole cell images, the Z-stack analysis (Figure 3C) showed no internalization of the probe, suggesting no active uptake of ManCous. Overall, the lack of inhibition from glucose in conjunction with the lack of uptake observed with HepG2 cells supports the GLUT5 specificity of ManCous. 

### 3.2. Profiling Fructose Uptake Efficiency and GLUT5 in Cells for Cancer Detection

We analyzed ManCou uptake in normal and cancer cell lines to evaluate the feasibility of cancer cell detection on the basis of the probe uptake through GLUT5. The comparative analysis of normal MCF10A, adenocarcinoma MCF7, and premalignant MCF10AneoT (ANeoT) cells was carried out through imaging. After treating cells with 20 µM concentration of ManCous for 10 min, the accumulated fluorescence was recorded and fluorescence intensity analyzed. We have observed minimal to no accumulation of probes in the normal MCF10A breast cells (comparable to that in HepG2 cells). The ManCous have excitation and emission maxima of 405 nm and 465 nm, respectively. The fluorescent images were captured in the blue channel. 

**Figure 5:**
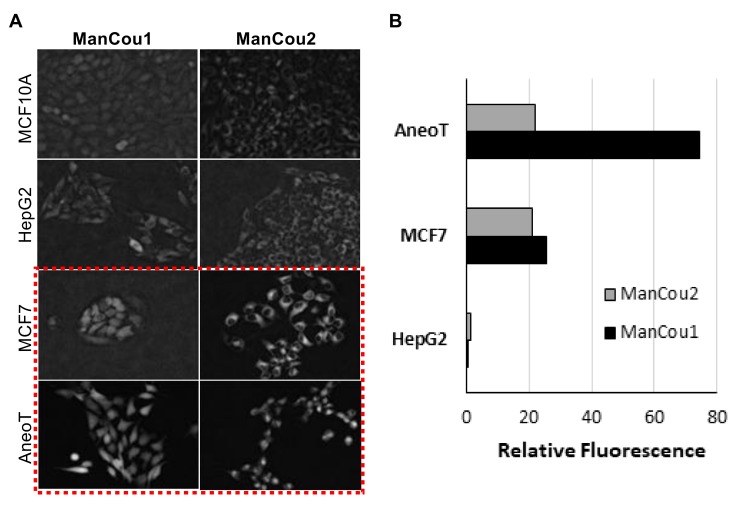
ManCou uptake in cells. (**A**) Grayscale fluorescence images of ManCou uptake in cells used to determine relative fluorescence using EVOS FL Auto at 20×; (**B**) relative differences in ManCou uptake (normalized for MCF10A cells). Data obtained for normal breast cells MCF 10A, hepatocellular cancer cells HepG2 (negative control), premalignant breast cancer cells AneoT, and breast adenocarcinoma cells MCF7 after 10 min of incubation with 20 µM of ManCous1 and 2. The *t*-tests (*p* < 0.05) performed after F-test (F ≤ Fcritical one-tail). For ManCou1, MCF7 was statistically significant compared to AneoT, HepG2, and MCF10A, while AneoT was statistically significant compared to HepG2 and MCF10A. For ManCou2, only MCF7 was statistically significant compared to AneoT, HepG2, and MCF10A.

**Figure 6:**
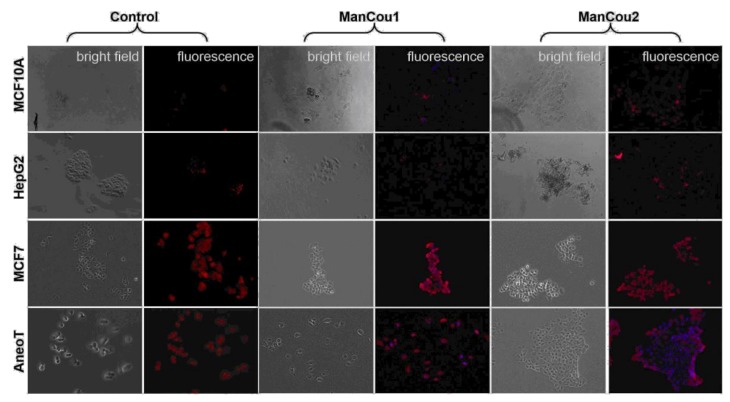
Immunostaining of cells for GLUT5 without and with ManCous. Optical microscopy, 20×. “Control” panel represents GLUT5 staining. “ManCou1” and “ManCou2” panels represent GLUT5 staining after 10 min treatment of cells with the respective probes. The red fluorescence represents GLUT5 (Texas Red^®^. Ex: 596 nm, Em: 620 nm) and blue (DAPI) represents the probe. The cells were incubated with 20 µM of the respective probes for 10 min and fixed in paraformaldehyde before immunostaining. Images were captured using EVOS FL Auto immediately following immunostaining.

As indicated by the grayscale fluorescent images in Figure 5A, the level of probe-induced fluorescence in the normal MCF10A cells is comparable to that of the HepG2 cells. The background fluorescence appears to originate from the residual association of probes with the membrane, as no fluorescence could be detected inside of MCF10A or HepG2 cells by Z-stack analysis. Thus, the data implicates that the uptake of ManCou probes in MCF7 and AneoT cells proceeds through GLUT5, resulting in a facile imaging of cancer cells. This was further confirmed by immunostaining the cells treated with probes for GLUT5, as shown in Figure 6. It is important to note that fructose uptake in the healthy tissues is limited to the liver and intestines, and fructose-specific GLUT5 is upregulated in cancer cells [23,24,30,44,45], supporting our approach to using fructose-based detection. This was further confirmed by immunostaining the cells treated with probes for GLUT5, as shown in Figure 6. 

**Figure 7:**
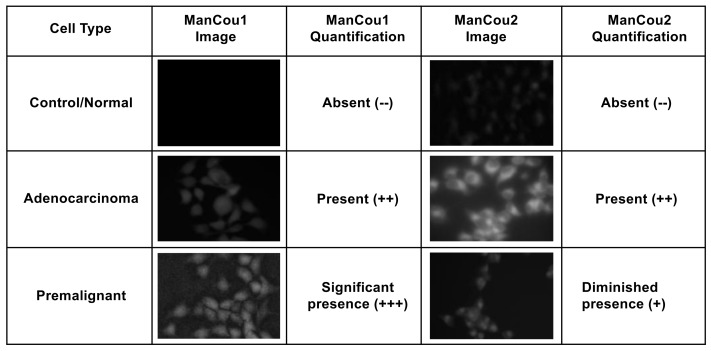
An example two-point analysis demonstrating the identification of normal, adenocarcinoma, and premalignant cells.

Using the normalized average CTCF with respect to the control MCF10A, the relative levels for each of the cells lines and treatment conditions can be directly assessed. As shown in Figure 5B, the fluorescence intensity observed for AneoT treated with ManCou1 was nearly 70 times higher than that for MCF10A and HepG2 cells for the same concentration and incubation period. Under the same conditions, AneoT had nearly seven times higher response compared to MCF7, clearly distinguishing the premalignant AneoT from the adenocarcinoma cells MCF7 (statistically significant data). Considering the plausible connection between the uptake of ManCou1 and the metabolic activity of the cells, the three-fold difference I the uptake between MCF7 and AneoT could reflect the higher metabolic activity of premalignant cells. Thus, the differences in the uptake of ManCou1 provides a one-point discrimination between normal and cancer cells. Under identical conditions, the ManCou2 probe had nearly 20 times higher uptake in the MCF7 than HepG2 cells. In contrast to ManCou1, ManCou2 is taken up at the relatively same levels in MCF7 and AneoT cells, suggesting similar levels of GLUT5 in both cell lines. Thus, the differences in the uptake of ManCou2 provide a second point for discriminating between cancer and normal cells. The cumulative analysis of ManCou1 and ManCou2 uptake reflecting the differences in GLUT levels and uptake efficiency also adds to a better discrimination between cancer subtypes. The expectations would be that in heterogeneous populations, treating cells with ManCou1 and ManCou2 and analyzing the CTCF will provide a visual discrimination of normal, premalignant, and adenocarcinoma cells. The two-point analysis would take the form of the analysis shown in Figure 7. The disparity in the signal makes this approach also ideal for spectrophotometric analysis in microplate or flow formats.

Reported evidence suggests that HepG2 cells lack the fructose-specific transporter GLUT5 [40], while breast cancer cells express it in significant levels. In order to assess the relation between uptake of the probe and the presence of the fructose-specific transporter GLUT5 in the cells tested, we carried out immunostaining of the cells for GLUT5 (Texas Red^®^. Ex: 596 nm, Em: 620 nm) following a 10-min incubation with 20 µM of the respective probes. Cells without the probes were used as controls. The differential expression of GLUT5 in the cellular membrane is evident from the GLUT5 immunostaining depicted in Figure 6 (“control” panel). As can be seen, there is little to no GLUT5 in MCF10A and HepG2 cells. In contrast, MCF7 cells and AneoT cells presented detectable levels of GLUT5. The lack of probe uptake (blue fluorescence) in MCF10A and HepG2 is represented well by the absence of GLUT5. Similarly, the probe uptake in MCF7 and AneoT cells are accompanied by the presence of GLUT5 (Figure 6, “ManCou” panels). The differences in GLUT5 levels appear to parallel the differences in the accumulation of ManCou probes in the normal and cancer cells. Overall, the ManCou uptake correlates with the presence of GLUT5, providing further evidence for the GLUT5-directed visualization of cancer cells. Further detailed analysis of GLUT5 expression and its relation to fructose will be required to develop a better understanding of the relationship between the fructose-specific transporters and uptake of the fructose-like probes. 

The outcomes of two-point analysis targeting GLUT5 are summarized in Figure 7. Overall, the statistical differences in the uptake of fluorescent probes ManCou1 and ManCou2 are statistically different, providing basis for clear discrimination between normal and cancer cells as well as between cancer subtypes. The correlations observed for the uptake of the ManCous and the presence of GLUT5 as well as significant differences in accumulation of the ManCous in cancer cells empowers optical screening of breast cancer using the fluorescent-labeled fructose analogs in multiplate or flow settings. Further analysis and experimentation is needed to quantify the relative abundance of GLUT5 to establish the correlations with ManCou uptake, as well as to evaluate the contribution from the cell population densities in a given sample to improve the detection system and hence the diagnosis. Combined with the fluorescent response, the two-point, two-metric system has the potential to clearly discriminate cancer cells and its subtypes. Future work will involve screening heterogeneous cultures derived from breast cancer biopsy samples for advancing the approach to POC technology.

## 4. Conclusions

We have successfully demonstrated the effective discrimination of premalignant and adenocarcinoma cancer cells using fluorescently labeled fructose analogs in vitro. The rapid response from the 10-min incubation and the observably different response between the cancer cells with the two fructose analogs highlight the use of this technique in high-throughput point-of-care healthcare applications. 

## 5. Patents

A provisional patent has been filed for the fluorescent fructose analog probes.

## Appendix A

Culture mediums used for the individual cell lines were formulated based on the recommended culture conditions from ATCC. The MCF 10A cells were cultured in mammary epithelial growth medium with the supplement kit from Lonza/Clonetics. Additionally, 100 ng/mL cholera toxin was added. The gentamicin supplement from the kit was not used as recommended. The MCF7 cells were cultured in Eagle’s minimum essential medium (EMEM) supplemented with 0.01 mg/mL human recombinant insulin and fetal bovine serum (FBS) to a final concentration of 10%. HepG2 was cultured in EMEM with 10% FBS. The MCF10AneoT cells were cultured in high-calcium DMEM/F12 (1:1) from Gibco with 1.05 mM CaCl_2_, 10 mM HEPES, 10 µg/mL insulin, 20 ng/mL EGF, 0.5 µg/mL hydrocortisone, 5% horse serum, and 100 ng/mL cholera toxin.
